# Validation of the dutch version of the health education impact questionnaire (HEIQ) and comparison of the Dutch translation with the English, German and French HEIQ

**DOI:** 10.1186/s12955-017-0601-4

**Published:** 2017-01-31

**Authors:** Judy W. Ammerlaan, Harmieke van Os-Medendorp, Jacob K. Sont, Gerald R. Elsworth, Richard H. Osborne

**Affiliations:** 1 Department Rheumatology and Clinical Immunology, University Medical Center Utrecht, HPN D02.244, PO Box 85500, 3508 GA Utrecht, The Netherlands; 2 Department Dermatology and Allergology, University Medical Center Utrecht, HPN D02.244, PO Box 85500, 3508 GA Utrecht, The Netherlands; 3 Leiden University Medical Center, Department of Medical Decision Making, J10-86, P.O. Box 9600, 2300 RC Leiden, The Netherlands; 4 Health Systems Improvement Unit, School of Health and Social Development, Deakin University, Geelong, 3220 Australia

**Keywords:** Chronic disease, Self-management, Health education, Questionnaire, heiQ, Validation, Translation, Comparison

## Abstract

**Background:**

The Health Education Impact Questionnaire (heiQ) evaluates the effectiveness of health education and self-management programs provided to people dealing with a wide range of conditions. Aim of this study was to translate, culturally adapt and validate the Dutch translation of the heiQ and to compare the results with the English, German and French translations.

**Methods:**

A systematic translation process was undertaken. Psychometric properties were studied among patients with arthritis, atopic dermatitis, food allergy and asthma (*n* = 286). Factorial validity using confirmatory factor analysis, item difficulty (D), item remainder correlation and composite reliability were conducted. Stability was tested using the intra-class correlation coefficient (ICC).

**Results:**

Items were well understood and only minor language adjustments were required. Confirmatory fit indices were >0.95 and item difficulty was D ≥ 0.65 for all items in scales showing acceptable fit indices, except for the reversed Emotional distress scale. Composite reliability ranged between 0.67 and 0.85. Test-retest reliability (*n* = 93) ICC varied between 0.61 and 0.84. Comparisons with other translations showed comparable fit indices. A lower ICC on Self-monitoring and insight scale was observed.

**Conclusions:**

The Dutch translation of the heiQ was found to be well understood and user friendly by patients with Rheumatoid Arthritis, Atopic Dermatitis, Food allergy and asthma and to have robust psychometric properties for evaluating the impact of health education and self-management programs. Given the wide applications of the heiQ and the comparability of the Dutch results with the English, German and French version, the heiQ is a practical and useful questionnaire to evaluate the impact of self-management support programs in different countries and populations with different diseases.

## Background

Although several new treatment options for people with chronic conditions like arthritis and atopic dermatitis have been developed in the past decade, patients still experience a large impact of their disease on their daily life [1–5]. The chronic nature of the disease imposes daily challenges and patients must make many decisions about the way they manage their lives [6–9]. It is not a matter of whether patients self-manage their (chronic) illness, but how they do this [10]. An individual’s ability to manage the symptoms, treatment, physical and psycho-social consequences and life style changes inherent in living with a chronic condition’ is often defined as self-management [11]. Over the past decade, several interventions have been developed to improve the self-management of chronically ill patients [12, 13]. While the initial aim of these interventions was to increase patient’s knowledge so they were able to change their behaviour [12, 14], the evidence subsequently demonstrated that increased knowledge was not enough. Other theoretical approaches, mostly derived from behavioural sciences, encouraged the movement of self-management interventions towards inclusion of cognitive-behavioural and other approaches [14].

One of the most studied self-management programs, based on Bandura’s self-efficacy theory, is the Chronic Disease Self- Management Program (CDSMP) of Stanford University, led by lay people to help people with a chronic disease gain confidence in their ability to control the symptoms and impact of their condition on their lives [12]. These programs generated great interest and numerous follow-up studies and government programs ensued [12, 15]. With the growing opportunities and use of the Internet, online self-management programs for patients with long-term conditions were developed [16, 17]. In an uncontrolled longitudinal evaluation of the online CDSMP program, at 12 months small to moderate improvements in health distress, fatigue and pain and self-efficacy were observed. In asthma, it has been shown that online self-management support results in a sustained improvement in disease control and asthma related quality of life [18]. This illustrates that self-management interventions can improve quality of life and well-being but their outcomes are varied and include measures of physical, psychological and social well-being [11, 14, 19]. Consequently, the diversity of patient populations, as the different theoretical foundations of the self-management interventions as well as the widely varying educational content of health education programs, make it challenging to demonstrate effectiveness with common metrics [14, 19–21].

In response to this issue, Osborne et al. [22] developed the Health Education Impact Questionnaire (heiQ) in Australia. The purpose of the heiQ is to provide a direct and realistic measurement of the impact and quality of self-management programs across settings and disease groups and also to provide highly relevant information on the outcomes of self-management programs to health professionals, policymakers and researchers. The development of the heiQ involved a grounded approach rather than a theory-based approach. Techniques used included review of current practice, development of a Program Logic, Concept Mapping, and rigorous item development based on the daily language of patients, and extensive item testing and validation in an independent sample. During the development, a wide range of stakeholders were involved, including; patients, health professionals, course leaders, academics and policy makers [22]. The original English heiQ [22] contained 42 items across eight independent scales: *Positive and active engagement in life (*five items*); Health directed activity (*four items*); Skill and technique acquisition (*five items*); Constructive attitudes and approaches (*five items*); Self-monitoring and insight (*seven items*); Health service navigation (*five items*); Social integration and support (*five items*)* and a reversed scale, *Emotional distress* (formally named Emotional wellbeing), (six items). The Cronbach’s alpha of the eight scales varied between *α =* 0.70 (Self-monitoring and insight) and *α* = 0.89 (Emotional distress). Higher values in the heiQ scales indicate better status, except for Emotional distress, where higher values indicate higher distress. Results from additional analysis showed that two items could be removed without compromising the content validity of the questionnaire [23]. A recent study on factor structure and measurement invariance of this latest version, confirmed these results [24]. Therefore, the revised English heiQ was shortened to 40 items with a 4 point response scale (strongly disagree, disagree, agree and strongly agree) [24]. The heiQ has been translated and validated into many languages including German [23] and French [25]. Both versions were translated and culturally adapted using forward and back translation, comprehensibility and content validity testing using interviews (German version) and committee review (French version). The psychometric properties of the German version were studied among 1202 adult patients with a range of chronic diseases from rehabilitation hospitals and the French version was studied among 1030 adult patients with renal failure, diabetes and arthritis. This group was randomly sampled from a health database. Psychometric analysis on reliability, factorial validity and concurrent validity of both the German and French heiQ were consistent with the original English version. The diversity of the patient population and cultural differences may affect patient’s perception about the instrument items [25]. As Morita et al. [26] showed in the validation study of the Japanese translation of the heiQ, in order to bridge the gap between the cultural differences between the Australian and Japanese subjects, the authors added examples to questionnaire items that Japanese people felt comfortable with.

In addition to existing online self-management programs, e.g., in asthma, the UMC Utrecht developed in close cooperation with their patient partners, four online self-management programs that aimed to improve an individual’s ability to cope with the symptoms, treatment and consequences of having a chronic disease like atopic dermatitis (AD), food allergy (FA), juvenile idiopathic arthritis (JIA) and arthritis and are developed for different target and age groups like young adults, adults and parents of young patients [27, 28]. Until now a questionnaire to evaluate the effectiveness of programs across chronic diseases has not been available. The aim for this study was to translate and culturally adapt the heiQ into Dutch to confirm the acceptability of the items and clarity of wording and then examine its construct validity in order to replicate the factor structure of the original heiQ. Next to that, stability and reliability was tested through stringent psychometrics. Subsequently, the validity and reliability of the Dutch heiQ was compared with the original English, German and French version heiQ.

## Methods

The study consisted of two steps. First, the original heiQ was translated and culturally adapted into Dutch, following the Deakin University Protocol [29]. In the second step, factorial validity, difficulty (D), item remainder correlation, composite reliability and stability (test-retest) were determined. Ultimately, results of the factor structure and reliability of the Dutch translation were compared with results of the original English, revised English, German and French translations of the heiQ.

### Translation and cultural adaptation

The original English heiQ [22] was translated by official independent translators using a forward translation by a professional translator followed by a blind back translation. Therefor a symmetrical translation was used, aimed to stay close to the original language of the heiQ [29]. After that, the translation was discussed by three Dutch researchers (JA, HvO, JS) each with fluency in English and experience with the content of questionnaire’s constructs, and also the original developer of the questionnaire, native English speaker (RO) during comprehensive consensus meetings to achieve equivalence between the heiQ in the original language and the Dutch translation of the heiQ.

Each item was assessed to ensure that the intent was equivalent to the English version and consensus was reached about the translation. The forward and backward translation resulted in a discussion between the researchers and the developer considering the translation of the word ‘confidently’. In the Dutch culture, the word relates to the word ‘privacy’ where the item in the original questionnaire refers to confidence. Furthermore there was a discussion about the word ‘depressed’. Where depression refers in the Dutch language to a (severe) psychiatric state, the word depressed in the original heiQ refers to ‘feeling down’. The adapted Dutch heiQ was further tested using cognitive interviews in a convenience sample of adult patients with arthritis, atopic dermatitis or food allergy. Following completion of the questionnaire the respondents were asked about their understanding of the questions, acceptability and clarity of wording. All cognitive interview data were discussed to generate a final consensus for the Dutch version.

### Psychometric analyses of the Dutch heiQ

#### Design and procedure

Cross-sectional survey data were used to investigate validity and reliability and two-week follow-up data were obtained on a subset for test-retest reliability. Patients were invited to fill in the questionnaire online after providing informed consent. The Medical Ethics Research Committee (MERC) of the UMC Utrecht and the Leiden UMC declared that this study did not apply to the Medical Research Involving Human Subjects Act and confirmed that official approval was not required. The last step consisted of the comparison of the Dutch heiQ with published reports of original English, revised English, German and French versions.

#### Population

The study population consisted of adults with atopic dermatitis, food allergy, asthma or rheumatoid arthritis. Inclusion criteria were aged 18 or over; attending the outpatient department of the University Medical Centre (UMC) Utrecht the Netherlands, diagnosed with Atopic dermatitis (AD) or Food Allergy (FA) or Rheumatoid Arthritis (RA) or participating in an online self-management program for AD or FA or participating in an internet-based asthma self-management support program by primary care practices in the Leiden region [30] able to read Dutch and have access to Internet.

#### Sample size estimation

In this study, the heiQ, comprising eight scales (each with 4 to 6 items) was to be subjected to a range of psychometric tests, including confirmatory factor analysis (CFA) to examine how well the hypothesized eight scales fit the data. It has been found that CFA models using relatively small samples of 250–1000 with categorical or ordinal data perform well using the software Mplus [31]. We therefore planned to include at least 250 patients.

#### Psychometric parameters and thresholds

The factorial validity of the Dutch heiQ was investigated by fitting eight single-scale factor models to the data, calculating item-remainder correlations and fitting a full eight-factor model. Fit indices included; Comparative Fit Index (CFI), Tucker Lewis Fit Index (TLI) and Root Mean Square Error of Approximation (RMSEA) and Weighted Root Mean Square Residual (WRMR). Chi-Square Test for Model Fit was also used. We investigated the one-factor model, for each of the eight scales and loadings per item; and the eight-factor model for the total heiQ. We then calculated item Difficulty (D) and Composite Reliability (CR) of scale/individual items. Difficulty was defined as the proportion of positive responses to the item. Stability (test-retest) of the questionnaire was tested using the intra-class correlation co-efficient (ICC). Illness-related variables (duration, severity of complaints/illness, comorbidity) and demographic variables (age, gender, educational level marital status, and children) were also collected.

#### Data analyses

Confirmatory factor analyses were carried out for the one-factor models and the eight-factor model. The mean and variance-adjusted weighted least squares estimator (WLSMV), suitable for the analysis of ordered categorical data, was used for the CFA analyses of the one factor models. The eight-factor model was estimated with robust maximum likelihood (MLR). Difficulty (D) was calculated directly from data on the frequency of item responses while item remainder correlations, composite reliability and their standard errors (SEs) were calculated for each of the eight scales with Mplus code developed by Raykov and colleagues [32]. Following recommendations of Raykov and others [33–35], acceptable threshold values for CFI, TLI, RMSEA and WRMR were, respectively, >0.95, >0.95, <0.06 and < 1.0. If one factor models were found to have chi-square <0.01 and RMSEA >0.08, correlated residuals were added if suggested by the largest modification index. Acceptable loadings were at least 0.5, according to de Vet et al. [36]. Variation in D gives information on the proportion of respondents who agree or disagree with an item. Therefore a D ranging between 30 and 70% was considered ideal for a scale designed to measure across a relatively broad range of the underlying construct. Item remainder correlations with 95% CI were reported; low item remainder correlation indicated that the question does not fit well in the scale. Composite reliability for full scales without 1 item and for the full scale were computed, including 95% confidence interval; scores of >0.7 were regarded as acceptable [32, 37]. The ICC was computed to determine test-retest reliability, with ICC >0.7 regarded as acceptable.

Results of the factorial validity and reliability of the Dutch translation were compared with published results of the German [23] and English [22], revised English [24] and French [25] translations. For this comparison, the CFI, RMSEA and Chi-square of model fit, and the composite reliability were collated. Also the performance of eight-factor analyses of the four versions across CFI, TLI and RMSEA (if available) and the intra class correlation per domain of the Dutch and German translation were compared.

## Results

### Acceptability and comprehensibility of the Dutch translation

Patients with arthritis (*n* = 9), atopic dermatitis (*n* = 4) and food allergy (*n* = 3), 8 men and 8 women with a mean age of 42.5 years (sd 17.3; range 16–73), judged the content and comprehensibility of the Dutch version of the heiQ. Overall, they understood the items as intended and found them acceptable. They had some suggestions like adding specific activity examples for the Dutch population (like biking and swimming) on the Health directed behavior scale which resulted in minor changes.

### Psychometric analyses

#### Participants

A total 286 patients participated in the study; 72% were women, 25% (*n* = 71) had Atopic Dermatitis, 31% (*n* = 88) Rheumatoid Arthritis, 23% (*n* = 66) Food Allergy and 21% (*n* = 61) asthma. Mean age was 43 years (sd 16; range 18–83 years) (Table 1). Test–retest analyses were carried out with 93 patients, mean age 49 years (sd 17; range 18–83 years). This sample contained 69% woman of whom 12% had AD, 79% RA and 10% FA.

**Table 1:** Demographic and clinical characteristics of study populations

	Food allergy (FA)	Atopic dermatitis (AD)	Rheumatoid arthritis (RA)	Asthma	Total
*n*	66	71	88	61	286
Sex: Female n (%)	(77.3)	(64.8)	(69.3)	(83.3)	(72%)
Age: mean, (sd) in years	36.0 (12.5)	34.9 (15.3)	53.6 (15.1)	42.5 (13.7)	42.5 (16.3)
Other demographic data (n)	14	18	88	n.a.	120
Marital status					
Single	50%	33%	14%		21%
Married	50%	61%	75%		70%
Divorced	0%	0%	9%		7%
Widowed	0%	6%	2%		3%
Level of education^a^					
Low	14%	11%	33%		28%
Medium	21%	22%	32%		29%
High	64%	67%	32%		41%
Other	0%	0%	3%		3%
Duration of disease mean (sd) in years	15.8 (11.7)	27.3 (14.8)	13.3 (11.8)		15.7 (13.1)
Self-rated health score mean (sd)^c^	7.1 (1.4)	7.3 (1.2)	6.5 (1.6)		6.7 (1.6)
Asthma Control Questionnaire (ACQ) (sd)^d^				1.0 (0.8)	
Food allergy characteristics^b^					
Peanut	50%				
Nuts	73%				
Egg	12%				
Cow’s milk	15%				
Other animal products	11%				
Fruits & vegetables	68%				

^a^ *Low* primary school or lower vocational secondary education, intermediate general secondary education; *Middle* intermediate vocational education, higher general secondary education; *High* higher vocational education or university education

^b^Individuals may have more than one food allergy

^c^measured on a scale from 1–10 (1 (worst) to 10 (excellent)

^d^ACQ: plausible range 0–6, controlled asthma: 0–0.75, partly controlled asthma: 0.75–1.5, uncontrolled asthma: >1.5)

#### Confirmatory factor analysis

After initial analyses of the one factor models, correlated residuals were added in for five subscales: Positive and active engagement in life, Emotional distress, Self-monitoring and insight, Social integration and support and Health service navigation. This resulted in CFI of all scales being >0.95, showing acceptable fit indices; RMSEA ≤ 0.06 for four scales: Health directed activity, Self-monitoring and insight, Constructive attitude and approaches, Social integration and support and WRMR <1.0 (Table 2). In Table 3 standardized factor loadings with 95% CI of all tested models are shown. Loadings of most scales were >0.5. Two items on the Self-monitoring and insight scale had a lower factor loading in the modified one-factor model, item 3 loaded 0.33 and item 6 loaded 0.48. The RMSEA and the SRMR of the eight factor model indicated good fit. CFI and TLI were, respectively, 0.89 and 0.88.

**Table 2:** Results of confirmatory factor analyses of the one-factor and eight factor model of the Dutch version

	CFI	RMSEA	WRMR	TLI	Chi-square of Model Fit		
					Value	DF	*P*
One factor models							
Health directed activity	1.00	0.06	0.32	1.00	3.92	2.00	0.14
Positive and active engagement in life	0.97	0.15	0.94	0.95	38.57	5.00	0.00
additional analysis: heiQ15 WITH heiQ8;	*1.00*	*0.07*	*0.44*	*1.00*	*10.28*	*4.00*	*0.04*
Emotional distress	0.99	0.10	0.70	0.99	36.21	9.00	0.00
additional analysis: heiQ12 WITH heiQ4;	*1.00*	*0.09*	*0.56*	*0.99*	*25.20*	*8.00*	*0.00*
Self-monitoring and insight	0.87	0.16	1.28	0.78	75.91	9.00	0.00
*additional analysis:* heiQ17 WITH heiQ3; heiQ11 WITH heiQ6;	*0.99*	*0.06*	*0.49*	*0.98*	*13.01*	*7.00*	*0.07*
Constructive attitudes and approaches	1.00	0.06	0.38	1.00	9.70	5.00	0.08
Skill and technique acquisition	1.00	0.08	0.30	0.99	5.80	2.00	0.05
Social integration and support	0.98	0.15	0.82	0.97	38.35	5.00	0.00
additional analysis heiQ31 WITH heiQ28; heiQ35 WITH heiQ28;	*1.00*	*0.04*	*0.27*	*1.00*	*4.46*	*3.00*	*0.22*
Health service navigation	0.98	0.18	1.03	0.97	48.82	5.00	0.00
additional analysis heiQ33 WITH heiQ32	*1.00*	*0.09*	*0.47*	*0.99*	*12.97*	*4.00*	*0.01*
Eight-factor model	0.89	0.05	0.064	0.88	1257.77	712	0.00

Acceptable: CFI >0.95; RMSEA < 0.06; WRMR <1.0; TLI > .95

**Table 3:** Item Difficulty and Item remainder correlation

	Item Difficulty (D)	95% Confidence interval around D	Item remainder correlation C	95% Confidence interval around C	Standardized factor loadings original model	Standardized factor loadings modified model^a^	95% CI for factor loadings ^b^
Health-directed activity							
heiQ1	0.83	0.78–0.87	0.74	0.69–0.78	0.83		0.78–0.89
heiQ9	0.76	0.71–0.81	0.67	0.61–0.72	0.78		0.72–0.84
heiQ13	0.73	0.68–0.78	0.78	0.74–0.81	0.90		0.86–0.95
heiQ19	0.65	0.59–0.70	0.58	0.51–0.64	0.67		0.59–0.74
Positive and active engagement in life							
heiQ2	0.85	0.80–0.88	0.66	0.60–0.72	0.78	*0.79*	*0.72–0.86*
heiQ5	0.97	0.95–0.99	0.65	0.56–0.72	0.75	*0.77*	*0.68–0.86*
heiQ8	0.83	0.78–0.87	0.68	0.61–0.73	0.79	*0.70*	*0.62–0.78*
heiQ10	0.87	0.82–0.90	0.73	0.66–0.78	0.84	*0.88*	*0.82–0.94*
heiQ15	0.82	0.77–0.86	0.60	0.54–0.65	0.71	*0.61*	*0.52–0.70*
Emotional distress							
heiQ4	0.40	0.34–0.46	0.66	0.59–0.72	0.73	*0.69*	*0.63–0.76*
heiQ7	0.13	0.10–0.18	0.66	0.61–0.71	0.71	*0.71*	*0.65–0.78*
heiQ12	0.25	0.21–0.31	0.81	0.78–0.85	0.88	*0.86*	*0.82–0.90*
heiQ14	0.15	0.12–0.20	0.81	0.78–0.84	0.90	*0.90*	*0.87–0.94*
heiQ18	0.08	0.05–0.12	0.83	0.79–0.86	0.91	*0.91*	*0.88–0.94*
heiQ21	0.12	0.09–0.16	0.82	0.79–0.85	0.91	*0.91*	*0.88–0.94*
Self–monitoring and insight							
heiQ3	0.82	0.77–0.86	0.35	0.26–0.44	0.52	*0.33*	*0.20–0.46*
heiQ6	0.76	0.71–0.81	0.43	0.33–0.51	0.52	*0.48*	*0.38–0.58*
heiQ11	0.94	0.91–0.97	0.47	0.39–0.55	0.55	*0.51*	*0.38–0.64*
heiQ16	0.76	0.71–0.81	0.76	0.71–0.81	0.67	*0.74*	*0.66–0.81*
heiQ17	0.90	0.86–0.93	0.75	0.69–0.80	0.71	*0.60*	*0.52–0.68*
heiQ20	0.87	0.83–0.91	0.49	0.39–0.57	0.64	*0.70*	*0.62–0.78*
Constructive attitude and approaches							
heiQ27	0.96	0.93–0.98	0.75	0.68–0.80	0.84		0.80–0.88
*heiQ34*	0.92	0.88–0.95	0.74	0.70–0.78	0.84		0.79–0.88
heiQ36	0.91	0.87–0.94	0.77	0.74–0.80	0.87		0.83–0.91
heiQ39	0.92	0.88–0.94	0.79	0.75–0.83	0.90		0.86–0.93
heiQ40	0.90	0.85–0.93	0.59	0.54–0.64	0.67		0.60–0.74
Skill and technique acquisition							
heiQ23	0.69	0.63–0.74	0.62	0.57–0.67	0.71		0.64–0.77
heiQ25	0.84	0.79–0.88	0.78	0.73–0.83	0.93		0.90–0.96
heiQ26	0.86	0.81–0.89	0.72	0.67–0.77	0.84		0.80–0.89
heiQ30	0.80	0.75–0.85	0.66	0.62–0.70	0.77		0.71–0.84
Social integration and support							
heiQ22	0.89	0.85–0.92	0.72	0.68–0.76	0.80	*0.77*	*0.72–0.83*
heiQ28	0.83	0.78–0.87	0.69	0.63–0.73	0.79	*0.89*	*0.82–0.95*
heiQ31	0.71	0.66–0.76	0.68	0.62–0.74	0.80	*0.83*	*0.78–0.88*
heiQ35	0.91	0.87–0.94	0.77	0.73–0.80	0.86	*0.88*	*0.84–0.92*
heiQ37	0.83	0.78–0.87	0.72	0.68–0.76	0.79	*0.76*	*0.70–0.82*
Health service navigation							
heiQ24	0.92	0.88–0.95	0.73	0.67–0.78	0.86	*0.87*	*0.82–0.92*
heiQ29	0.91	0.87–0.94	0.69	0.64–0.73	0.77	*0.79*	*0.73–0.84*
heiQ32	0.96	0.93–0.98	0.69	0.63–0.74	0.79	*0.70*	*0.62–0.77*
heiQ33	0.92	0.88–0.94	0.72	0.69–0.74	0.85	*0.78*	*0.72–0.84*
heiQ38	0.87	0.83–0.91	0.76	0.71–0.81	0.86	*0.89*	*0.85–0.93*

^a^standardized factor loadings of one-factor models (based on the models with correlated residuals as in Table 2)

^b^95% confidence interval of original model (set roman/right up) or of the modified model in italics

#### Item difficulty, item remainder correlation and reliability

Results of item difficulty analysis for Health directed activity, Positive and active engagement in life, Skill and technique acquisition, Self-monitoring and insight, Social integration and support and the reversed scale Emotional distress indicated that the D of items ranged between 0.60 and 0.97 (Table 3). The scales Constructive attitudes and approaches and Health service navigation were found to have D with a smaller range, between 0.87 and 0.96, indicating that most answers on the items were located in the agree/strongly agree end of the scale. Item remainder correlation of items (Table 3) of all scales except Self-monitoring and insight were ≥ 0.58, indicating that all of the items hang together well as a scale. Item remainder correlation of items of Self-monitoring and insight varied between 0.35 and 0.76, indicating that there is less cohesion between the items. Composite reliability of the subscale Self-monitoring and insight was 0.67 (95% CI 0.61–0.73). For all other subscales composite reliability was ≥ 0.81. ICC varied between 0.61 and 0.84 (Table 4).

**Table 4:** Composite reliability and intra class correlation per domain

	Composite reliability ^a^	Confidence Interval	Intra class Correlation (*n* = 93)	95% Confidence Interval Lower Bound - Upper Bound
Health directed activity	0.82	0.79–0.86	0.84	0.77–0.89
Positive and active engagement in life	0.81	0.78–0.85	0.74	0.63–0.82
Emotional distress	0.89	0.87–0.91	0.86	0.80–0.91
Self-monitoring and insight	0.67	0.61–0.73	0.61	0.47–0.73
Constructive attitudes and approaches	0.86	0.83–0.89	0.69	0.57–0.78
Skill and technique acquisition	0.82	0.78–0.85	0.67	0.55–0.77
Social integration and support	0.85	0.83–0.88	0.73	0.62–0.81
Health service navigation	0.85	0.82–0.88	0.65	0.52–0.75

^a^ Acceptable: CR > 0.7

## Comparison of the Dutch heiQ (NL) with the German (G), English (ENG), English revised (ENG-R) and French heiQ translations

### Comparison of confirmatory factor analyses across language versions

Comparison of the one factor validation of the Dutch (NL), German (G) [23], English (ENG) [22] and English revised (ENG-R) [24] translations showed acceptable CFI for all translations. In most translations, the RMSEA with an acceptable value of <0.06, was adequate; except for Positive and active engagement in life (Dutch translation (0.07)), Emotional distress (Dutch (0.09), English (0.07) and English revised (0.07) translation), Health directed activity (English translation (0.09)), Skills and technique acquisition (Dutch translation (0.08)) and Health service navigation (Dutch translation (0.09)). The p-value of Chi square Model fit was <0.10; except for Health directed activity (Dutch translation (0.14)), Constructive attitudes and approaches (English translation (0.17)), Skills and techniques (German (0.59) and English translation (0.44)) and Health service navigation (English translation (0.44)).

### Comparison of composite reliability across language versions

Composite reliability was acceptable for the Dutch, German and English translations except for the domain Self-monitoring and insight of the Dutch translation (see Table 5). The reliability of this domain was also relatively low for the English translation (0.70). The reliability of the French translation ranged from 0.74 to 0.89 [25].

**Table 5:** Results of the composite reliability of the Dutch, German, English (original) and English (revised) versions of the heiQ

	Dutch	German	English (original)^c^	English (revised)
Health directed activity	0.82	0.83	0.80	0.83
Positive and active engagement in life	0.81	0.75	0.86	0.83
Emotional distress	0.89	0.88	0.89	0.86
Self-monitoring and insight ^a^	0.67	0.74	0.70^a^	0.74
Constructive attitude and approaches	0.86	0.87	0.81	0.88
Skills and techniques acquisition^b^	0.82	0.77	0.81^b^	0.80
Social integration and support	0.85	0.88	0.86	0.88
Health service navigation	0.85	0.87	0.82	0.85

Acceptable: Composite reliability >0.7

^a^The English heiQ (original) Self-monitoring and insight scale contained 7 items (including ‘I know when my lifestyle is creating health problems for me)

^b^The English heiQ (original) Skill and technique acquisition scale contains 5 items (including ‘I have effective skills that help me handle stress’)

^c^Cronbach’s alpha was used in the original English heiQ analysis

### Comparison of eight factor models across language versions

Comparison the eight factor model of the Dutch, English, German and French translations showed similar results for CFI and RMSEA (see Table 6)

**Table 6:** Comparison of the CFI, TLI and RMSEA of the Dutch, German, English and French versions of the heiQ, eight factor model

CFA model	CFI	TLI	RMSEA
Dutch	0.89	0.88	0.05
German	0.92	n.a	0.04
English	0.99	n.a	0.04
French	0.92	0.91	0.04

Acceptable: CFI >0.95; TLI > .95; RMSEA < 0.06

### Comparison of Intra Class Correlation (ICC) across language versions

For the comparison of the ICC, only the results of the Dutch and German translation were available. The Dutch and German translations had an ICC <0.7 on the scales Self-monitoring and insight and Health service navigation. The German translation also had ICC <0.7 on Health directed activity, while the Dutch translation had an ICC <0.7 on Constructive attitude and approaches and Skills and techniques acquisition (Table 7).

**Table 7:** Comparison of the intra class correlation per domain for the Dutch and German version of the heiQ

	Dutch *N* = 93	German *N* = 69
Health directed activity	0.84	0.60
Positive and active engagement in life	0.74	0.72
Emotional distress	0.86	0.77
Self-monitoring and insight	0.61	0.63
Constructive attitude and approaches	0.69	0.77
Skills and techniques acquisition	0.67	0.72
Social integration and support	0.73	0.83
Health service navigation	0.65	0.68

Acceptable: ICC > 0.7

## Discussion

In this study, the English heiQ was translated into the Dutch language and psychometric properties were determined. The results show that the Dutch heiQ has good psychometric properties in diverse groups of patients indicating that it is likely to be a robust outcomes measure of health education and self-management programs in The Netherlands. We also showed that psychometric properties of the Dutch translation were comparable with the English, German and French translations, which provides evidence that the heiQ conceptualizes a broad range of self-management skills in a consistent way across chronic conditions, cultures and languages.

Confirmatory Factor Analysis was used to investigate the factorial validity, showing acceptable fit indices for all eight scales of the Dutch translation of the heiQ. The RMSEA and the SRMR of the eight factor model indicated good fit, CFI and TLI were lower. It is known that CFI and TLI do not function well in correctly specified models with larger numbers of variables, while the RMSEA tends to improve in correctly specified models with large numbers of variables [38]. The data indicate that the eight factor structure is maintained across settings with different languages, cultures and healthcare systems.

There appears to be an appropriate level and range of difficulty for most scales; Health directed activity, Positive and active engagement in life, Emotional distress, Self-monitoring and insight, Skill and technique acquisition and Social integration and support: However, for Constructive attitudes and approaches and Health service navigation, a more restricted range of difficulties were found which may mean that these scales may not have strong discrimination between persons. Item difficulty is a reflection of the quality of items and the status of the people completing the questionnaire. In our sample, the majority of people answered the items with strongly agree. The respondents were relatively young and the average duration of living with their condition was over 10 years. It is likely that this construct, like many of them, changes greatly over time, and over the course of the disease the respondents in our study came to have a very good understanding of the healthcare system and have a constructive attitude. Further work on the heiQ is needed to explore change over time, including pathways from diagnosis to effective long term self-management.

Reliability of the subscales of the Dutch translation is acceptable. However the domain Self-Monitoring and insight has lower item remainder correlation and composite reliability (0.67) than other scales. Self-monitoring and Insight captures *‘the individuals’ ability to monitor their condition and their physical and or emotional responses that leads to insight and appropriate actions to self-manage*’. The relatively low reliability of this domain accords with the English and German versions. It is possible that this domain consists of two separate concepts: a) self-monitoring (check and action) and b) insight and understanding of the underlying disease processes. We also found relatively low factor loadings on three items in this domain (Table 3), indicating the limited relationship between the items and the latent construct. Future research is needed to identify if this domain could be redeveloped into two separate constructs.

Four domains showed test-retest interclass correlation scores of <0.7 (Self-monitoring and Insight (0.61), Constructive attitude and approaches (0.69), Skills and Technique (0.67) and Health service navigation (0.65)), indicating modest stability, and suggesting that these constructs are less stable over time. People with Rheumatoid Arthritis and Atopic Dermatitis do experience exacerbations and remissions over time [4, 5] which influences test-retest estimates. Test-retest was also examined in validation of the German translation, showing comparable results, except for two scales. For the Health directed activity scale the ICC of Dutch translation was 0.84, while the German translation had a substantially lower ICC of 0.60. The opposite was found for Social integration and support, where the ICC for the Dutch translation (0.73) was somewhat lower, compared with the German translation (0.83). Differences in these results could be due to differences in how the items were translated, cultural factors or the study population – regarding the latter; our study mostly comprised people with RA versus orthopedic patients in the German test-retest.

Translation of the original English version of the heiQ was carried out according to international standards, including a back translation and review by a multidisciplinary team, including the developer of the questionnaire. The aim of these standards is to achieve a questionnaire of which results could be compared across different languages. Epstein et al. [25] revealed that a multidisciplinary expert team is san critical contributor to psychometric properties. Importantly, in our study, we also used a carefully constructed item intent guidance document, and one of the authors of the heiQ (RO), who has chaired the translation of over 20 language versions of the heiQ. Comparison of the psychometric properties of the Dutch, German [23], English [22] and French [25] translation of the heiQ showed that most domains have good fit indices. The importance of good fit indices for, e.g., the one-factor models, is that they indicate greater scale unidimensionality (all items measuring the same construct). Unidimensionality is distinct from reliability and is, arguably, at least as important [39]. In addition, Brunet et al. [40] showed that 5 domains of the French- and English translation measured these domains in the same way across both language groups. The study population of this Dutch study consisted of people with chronic illnesses including Rheumatoid Arthritis, Atopic Dermatitis, Food allergy and asthma. Other validation studies of the eight factor heiQ among different patient groups like patients with metabolic syndromes [26] and patients with musculoskeletal disorders, psoriasis, Type ll diabetes and heart disease [41] also showed similar outcomes. Maunsell et al. [42] supported in their validation study among adults with cancer that five separated heiQ scales where also acceptable and valuable to evaluate empowerment support interventions. Considering the usefulness of the heiQ questionnaire to evaluate self-management programs, several studies have been published. Cheng et al. [43] evaluated the efficacy of a Chronic Disease Self-Management Program with the heiQ among people with a chronic disease in New Zealand. The results of this cross-sectional pre-posttest design showed improvement on seven of the eight subscales. Next to that, the effectiveness of an online self-management program in improving health outcomes and well-being for gay men living with HIV showed significant improvement on four subscales of the heiQ in the intervention group [44]. Given that the heiQ has been widely taken up across different settings and diseases [26, 41–43, 45] and psychometric analyses continue to provide a growing web of evidence that it is has robust properties, the questionnaire is likely to continue to have relevance for different stakeholders and support them in decision-making about the value and impact of self-management and health education programs.

Limitations of this study include the small sample size which made it not possible to run factorial invariances across the different patient’s groups. Next to that, as mentioned before concerning Item difficulty, the respondents of our study were relatively young and the average duration of disease was relatively long (≥10 years). More research with a larger sample per different chronic disease and variation on age and duration of disease is needed.

## Conclusion

The Dutch translation of the heiQ was found to be well understood by patients with Rheumatoid Arthritis, Atopic Dermatitis, Food allergy and asthma and to have robust psychometric properties for evaluating the impact of health education and self-management programs. Given the wide applications of the heiQ and the comparability of the Dutch results with the English, German and French version, we conclude that the heiQ is a practical and useful questionnaire to evaluate the impact of self-management support programs in different countries and populations with different diseases.

## Abbreviations

ACQ: Asthma control questionnaire
AD: Atopic dermatitis
CDSMP: Chronic disease self-management program
CFA: Confirmatory factor analysis
CFI: Comparative fit index
CI: Confidence interval
CR: Composite reliability
D: Item difficulty
ENG: English translation
ENG-R: English revised translation
FA: Food allergy
G: German translation
heiQ: Health education impact questionnaire
ICC: Intra-class correlation coefficient
JIA: Juvenile idiopathic arthritis
MERC: Medical Ethics Research Committee
MLR: Maximum likelihood
NL: Dutch translation
RMSEA: Root mean square error of approximation
SD: Standard deviation
SEs: Standard errors
TLI: Tucker lewis fit index
UMC Utrecht: University Medical Center Utrecht
WLSMV: Weighted least squares estimator
WRMR: Weighted root mean square residual

## References

[1] Kiebert G, Sorensen SV, Revicki D, Fagan SC, Doyle JJ, Cohen J, Fivenson D (2002). Atopic dermatitis is associated with a decrement in health-related quality of life. Int J Dermatol.

[2] Le TM, Flokstra-de Blok BM, van Hoffen E, Lebens AF, Goossens NJ, Dubois AE, Bruijnzeel-Koomen CA, Knulst AC (2013). Quality of life is more impaired in patients seeking medical care for food allergy. Int Arch Allergy Immunol.

[3] Peniamina RL, Bremer P, Conner TS, Mirosa M (2014). Understanding the needs of food-allergic adults. Qual Health Res.

[4] Eichenfield LF, Tom WL, Chamlin SL, Feldman SR, Hanifin JM, Simpson EL, Berger TG, Bergman JN, Cohen DE, Cooper KD, Cordoro KM, Davis DM, Krol A, Margolis DJ, Paller AS, Schwarzenberger K, Silverman RA, Williams HC, Elmets CA, Block J, Harrod CG, Begolka WS, Sidbury R. Guidelines of care for the management of atopic dermatitis: Section 1. Diagnosis and assessment of atopic dermatitis. J Am Acad Dermatol. 2013. 10.1016/j.jaad.2013.10.010 PMC4410183 24290431

[5] Overman CL, Jurgens MS, Bossema ER, Jacobs JW, Bijlsma JW, Geenen R (2014). Change of psychological distress and physical disability in patients with rheumatoid arthritis over the last two decades. Arthritis Care Res (Hoboken).

[6] Salaffi F, Carotti M, Gasparini S, Intorcia M, Grassi W (2009). The health-related quality of life in rheumatoid arthritis, ankylosing spondylitis, and psoriatic arthritis: a comparison with a selected sample of healthy people. Health Qual Life Outcomes.

[7] Kristiansen TM, Primdahl J, Antoft R, Horslev-Petersen K (2012). Everyday life with rheumatoid arthritis and implications for patient education and clinical practice: a focus group study. Musculoskeletal Care.

[8] Zuberbier T, Orlow SJ, Paller AS, Taieb A, Allen R, Hernanz-Hermosa JM, Ocampo-Candiani J, Cox M, Langeraar J, Simon JC (2006). Patient perspectives on the management of atopic dermatitis. J Allergy Clin Immunol.

[9] Hamnes B, Hauge MI, Kjeken I, Hagen KB (2011). ‘I have come here to learn how to cope with my illness, not to be cured’: a qualitative study of patient expectations prior to a one-week self-management programme. Musculoskeletal Care.

[10] Bodenheimer T, Lorig K, Holman H, Grumbach K (2002). Patient self-management of chronic disease in primary care. JAMA.

[11] Barlow J, Wright C, Sheasby J, Turner A, Hainsworth J (2002). Self-management approaches for people with chronic conditions: a review. Patient Educ Couns.

[12] Lorig KR, Holman H (2003). Self-management education: history, definition, outcomes, and mechanisms. Ann Behav Med.

[13] Newman S, Steed L, Mulligan K (2004). Self-management interventions for chronic illness. Lancet.

[14] Iversen MD, Hammond A, Betteridge N (2010). Self-management of rheumatic diseases: state of the art and future perspectives. Ann Rheum Dis.

[15] Osborne RH, Spinks JM, Wicks IP (2004). Patient education and self-management programs in arthritis. Med J Aust.

[16] Lorig KR, Ritter PL, Dost A, Plant K, Laurent DD, McNeil I (2008). The expert patients programme online, a 1-year study of an Internet-based self-management programme for people with long-term conditions. Chronic Illn.

[17] van der Meer V, Bakker MJ, van den Hout WB, Rabe KF, Sterk PJ, Kievit J, Assendelft WJ, Sont JK, SMASHING (Self-Management in Asthma Supported by Hospitals, ICT, Nurses and General Practitioners) Study Group (2009). Internet-based self-management plus education compared with usual care in asthma: a randomized trial. Ann Intern Med.

[18] van Gaalen JL, Beerthuizen T, van der Meer V, van Reisen P, Redelijkheid GW, Snoeck-Stroband JB, Sont JK, SMASHING Study Group (2013). Long-term outcomes of internet-based self-management support in adults with asthma: randomized controlled trial. J Med Internet Res.

[19] Trappenburg J, Jonkman N, Jaarsma T, van Os-Medendorp H, Kort H, de Wit N, Hoes A, Schuurmans M (2013). Self-management: one size does not fit all. Patient Educ Couns.

[20] Nolte S, Elsworth GR, Newman S, Osborne RH (2013). Measurement issues in the evaluation of chronic disease self-management programs. Qual Life Res.

[21] Kroon FP, van der Burg LR, Buchbinder R, Osborne RH, Johnston RV, Pitt V (2014). Self-management education programmes for osteoarthritis. Cochrane Database Syst Rev.

[22] Osborne RH, Elsworth GR, Whitfield K (2007). The Health Education Impact Questionnaire (heiQ): an outcomes and evaluation measure for patient education and self-management interventions for people with chronic conditions. Patient Educ Couns.

[23] Schuler M, Musekamp G, Faller H, Ehlebracht-Konig I, Gutenbrunner C, Kirchhof R, Bengel J, Nolte S, Osborne RH, Schwarze M (2013). Assessment of proximal outcomes of self-management programs: translation and psychometric evaluation of a German version of the Health Education Impact Questionnaire (heiQ). Qual Life Res.

[24] Elsworth GR, Nolte S, Osborne RH. Factor structure and measurement invariance of the Health Education Impact Questionnaire: Does the subjectivity of the response perspective threaten the contextual validity of inferences? SAGE Open Med. 2015;3. 10.1177/2050312115585041 PMC4679238 26770785

[25] Epstein J, Osborne RH, Elsworth GR, Beaton DE, Guillemin F (2015). Cross-cultural adaptation of the health education impact questionnaire: experimental study showed expert committee, not back-translation, added value. J Clin Epidemiol.

[26] Morita R, Arakida M, Osborne RH, Nolte S, Elsworth GR, Mikami H (2013). Adaptation and validation of the Japanese version of the Health Education Impact Questionnaire (heiQ-J) for the evaluation of self-management education interventions. Jpn J Nurs Sci.

[27] van Os-Medendorp H, van Leent-de Wit I, de Bruin-Weller M, Knulst A (2015). Usage and users of online self-management programs for adult patients with atopic dermatitis and food allergy: an explorative study. JMIR Res Protoc.

[28] Ammerlaan J, van Os-Medendorp H, Scholtus L, de Vos A, Zwier M, Bijlsma H, Kruize AA (2014). Feasibility of an online and a face-to-face version of a self-management program for young adults with a rheumatic disease: experiences of young adults and peer leaders. Pediatr Rheumatol Online J.

[29] Hawkins M, Osborne RH. Questionnaire translation and cultural adaption procedure version 1.0. 2010.

[30] van Gaalen JL, Bakker MJ, van Bodegom-Vos L, Snoeck-Stroband JB, Assendelft WJ, Kaptein AA, van der Meer V, Taube C, Thoonen BP, Sont JK, IMPASSE study group (2012). Implementation strategies of internet-based asthma self-management support in usual care Study protocol for the IMPASSE cluster randomized trial. Implement Sci.

[31] Beauducel A, Herzberg PY (2006). Performance of maximum likelihood versus means and variance adjusted weighted least squares estimation in CFA. Struct Equ Model Multidiscip J.

[32] Raykov T, Marcoulides GA (2011). Introduction to psychometric theory.

[33] Hu L, Bentler PM (1999). Cutoff criteria for fit indexes in covariance structure analysis: conventional criteria versus new alternatives. Struct Equ Model Multidiscip J.

[34] Browne MW, Cudeck R, Bollen KA, Long JS (1993). Alternative ways of assessing model fit. testing structural equation models.

[35] Raykov T. On the large-sample bias, variance, and mean squared error of the conventional noncentrality parameter estimator of covariance structure models. Struct Equation Modeling. 2000;7:431–41.

[36] De Vet HCW, Terwee CB, Mokkink LB, Knol D (2015). Chapter 4. Field-testing: item reduction and data structure. Measurement in medicine.

[37] Raykov T, Hoyle RH (2012). Scale construction and development using structural equation modeling. Handbook of structural equation modeling.

[38] Kenny DA, McCoach DB (2003). Effect of the number of variables on measures of fit in structural equation modeling. Struct Equ Model.

[39] Hattie JA (1985). Methodology review: assessing unidimensionality of tests and items. Appl Psychol Meas.

[40] Brunet J, Lauzier S, Campbell HS, Fillion L, Osborne RH, Maunsell E (2015). Measurement invariance of English and French Health Education Impact Questionnaire (heiQ) empowerment scales validated for cancer. Qual Life Res.

[41] Wahl AK, Osborne RH, Langeland E, Wentzel-Larsen T, Mengshoel AM, Ribu L, Peersen K, Elsworth GR, Nolte S (2016). Making robust decisions about the impact of health education programs: psychometric evaluation of the Health Education Impact Questionnaire (heiQ) in diverse patient groups in Norway. Patient Educ Couns.

[42] Maunsell E, Lauzier S, Brunet J, Pelletier S, Osborne RH, Campbell HS (2014). Health-related empowerment in cancer: validity of scales from the Health Education Impact Questionnaire. Cancer.

[43] Cheng JJ, Arenhold F, Braakhuis AJ (2016). Determining the efficacy of the chronic disease self-management programme and readability of ‘living a healthy life with chronic conditions’ in a New Zealand setting. Intern Med J.

[44] Millard T, Agius PA, McDonald K, Slavin S, Girdler S, Elliott JH (2016). The positive outlook study: a randomised controlled trial evaluating online self-management for HIV positive gay men. AIDS Behav.

[45] Osborne RH, Batterham R, Livingston J (2011). The evaluation of chronic disease self-management support across settings: the international experience of the health education impact questionnaire quality monitoring system. Nurs Clin North Am.

